# Supplementary figures and images for: The Serine Protease Autotransporters TagB, TagC, and Sha from Extraintestinal Pathogenic Escherichia coli Are Internalized by Human Bladder Epithelial Cells and Cause Actin Cytoskeletal Disruption
Source: Int J Mol Sci. 2020 Apr 26;21(9):3047. doi: 10.3390/ijms21093047 (PMC7246781; doi:10.3390/ijms21093047)

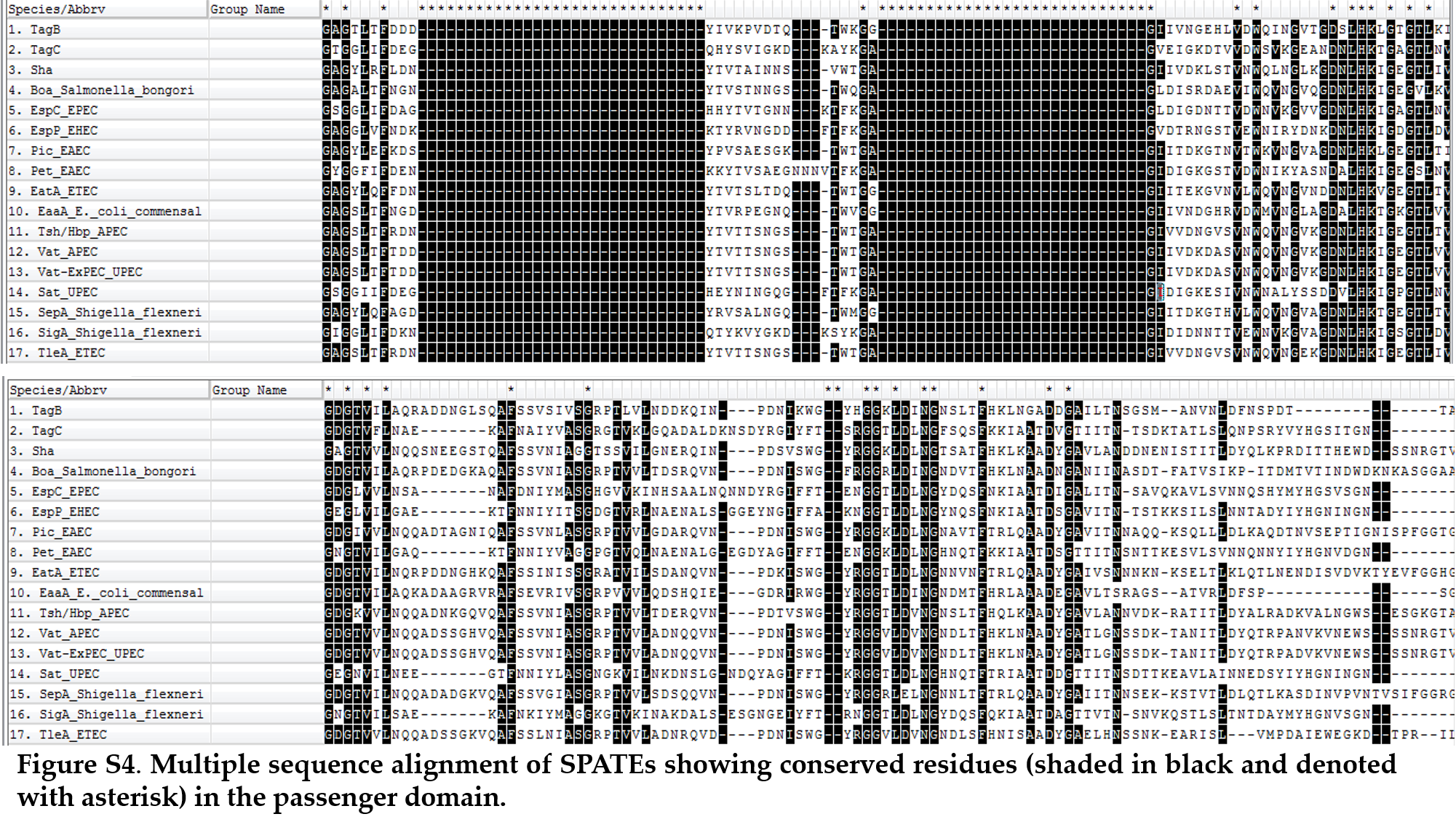

Supplement: Supplementary file 1 [file ijms-21-03047-s001.zip › Supplement Figure S4 II.tif]

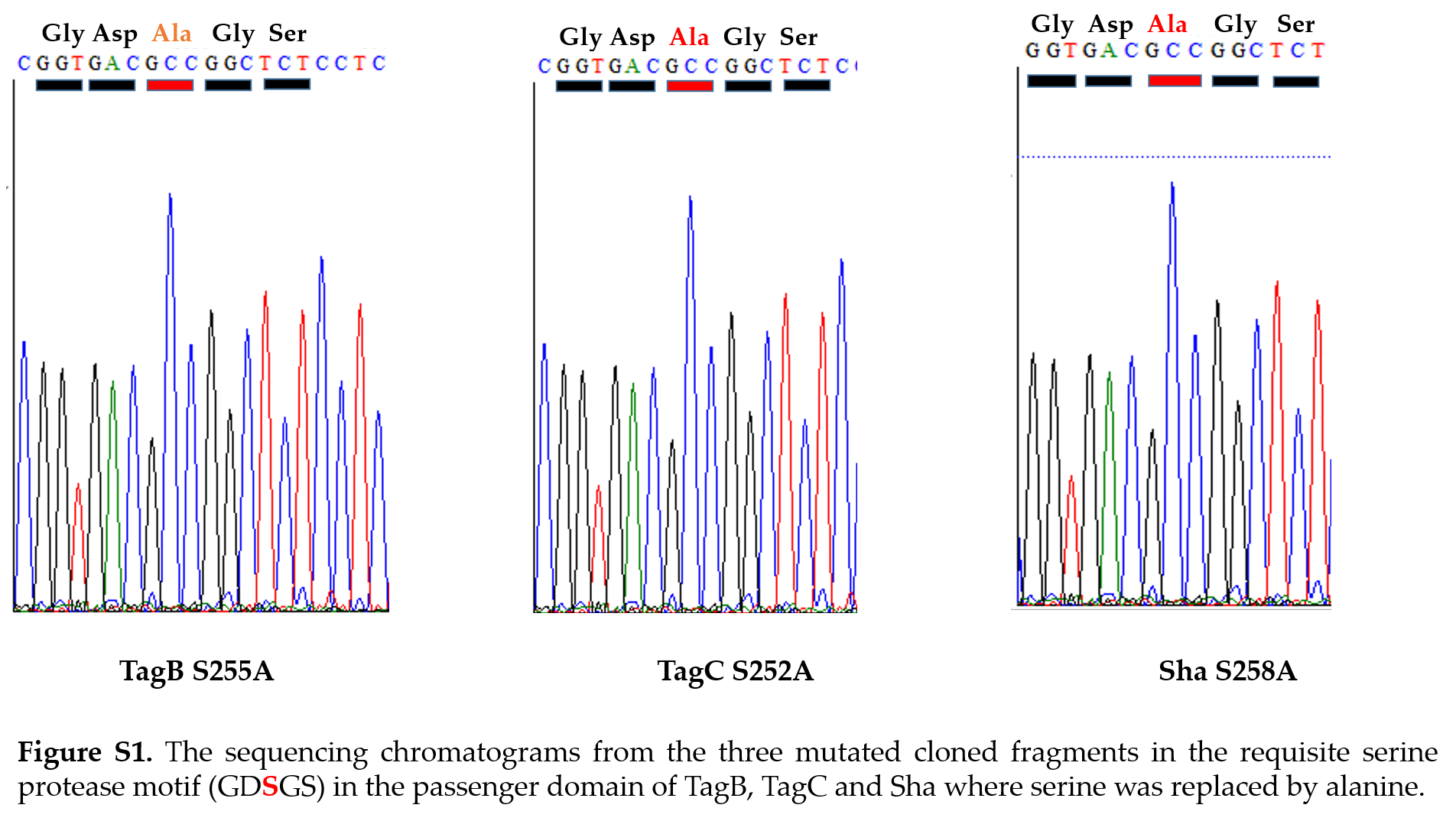

Supplement: Supplementary file 1 [file ijms-21-03047-s001.zip › Supplement Figure S1.tif]

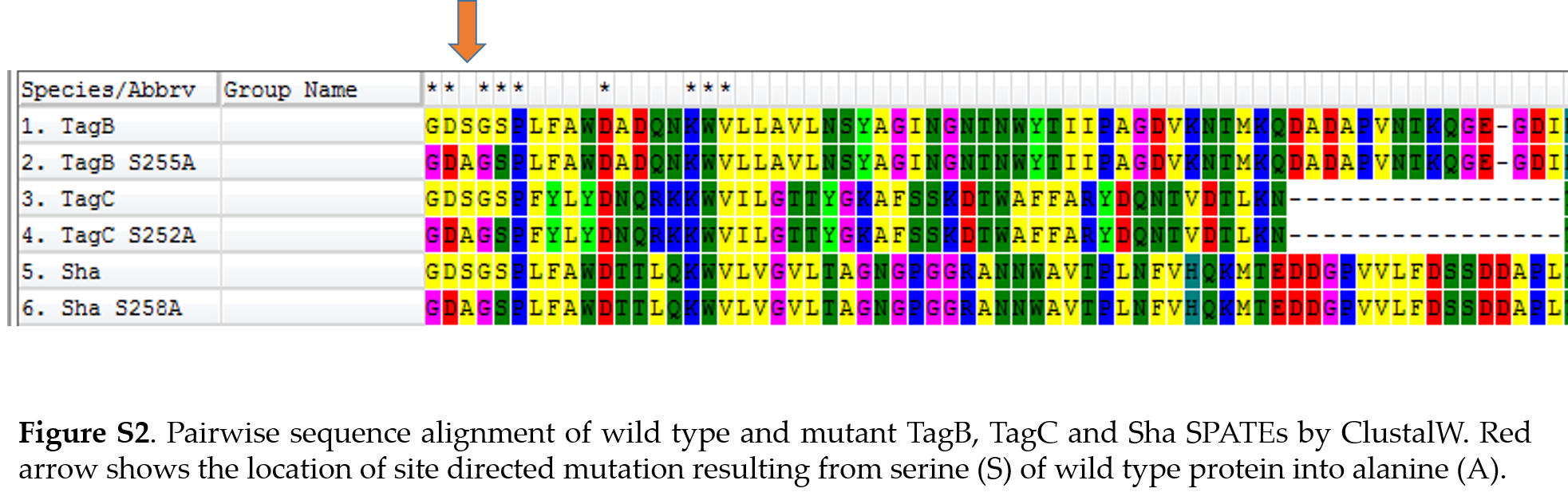

Supplement: Supplementary file 1 [file ijms-21-03047-s001.zip › Supplement Figure S2.tif]

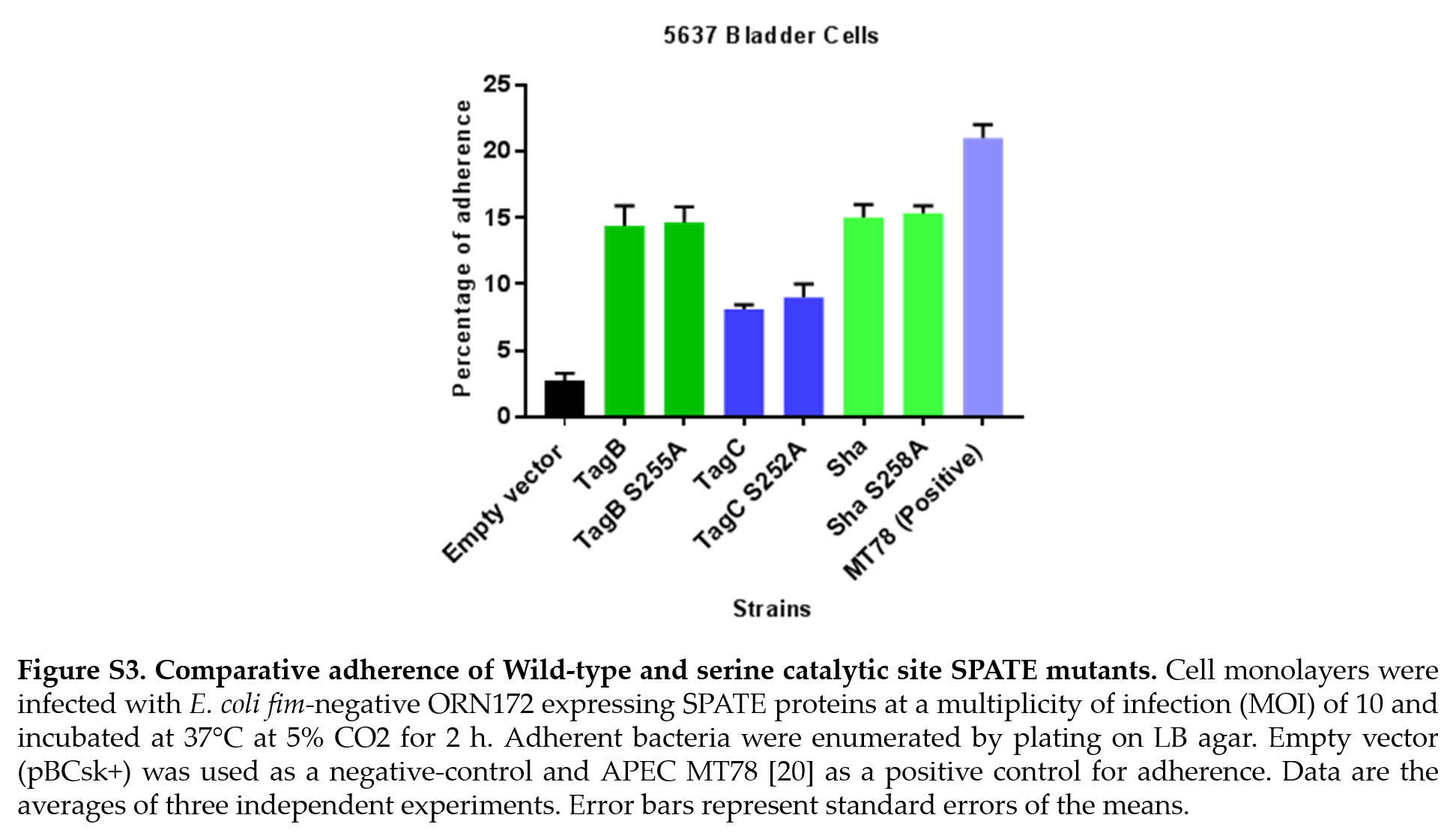

Supplement: Supplementary file 1 [file ijms-21-03047-s001.zip › Supplement Figure S3.tif]

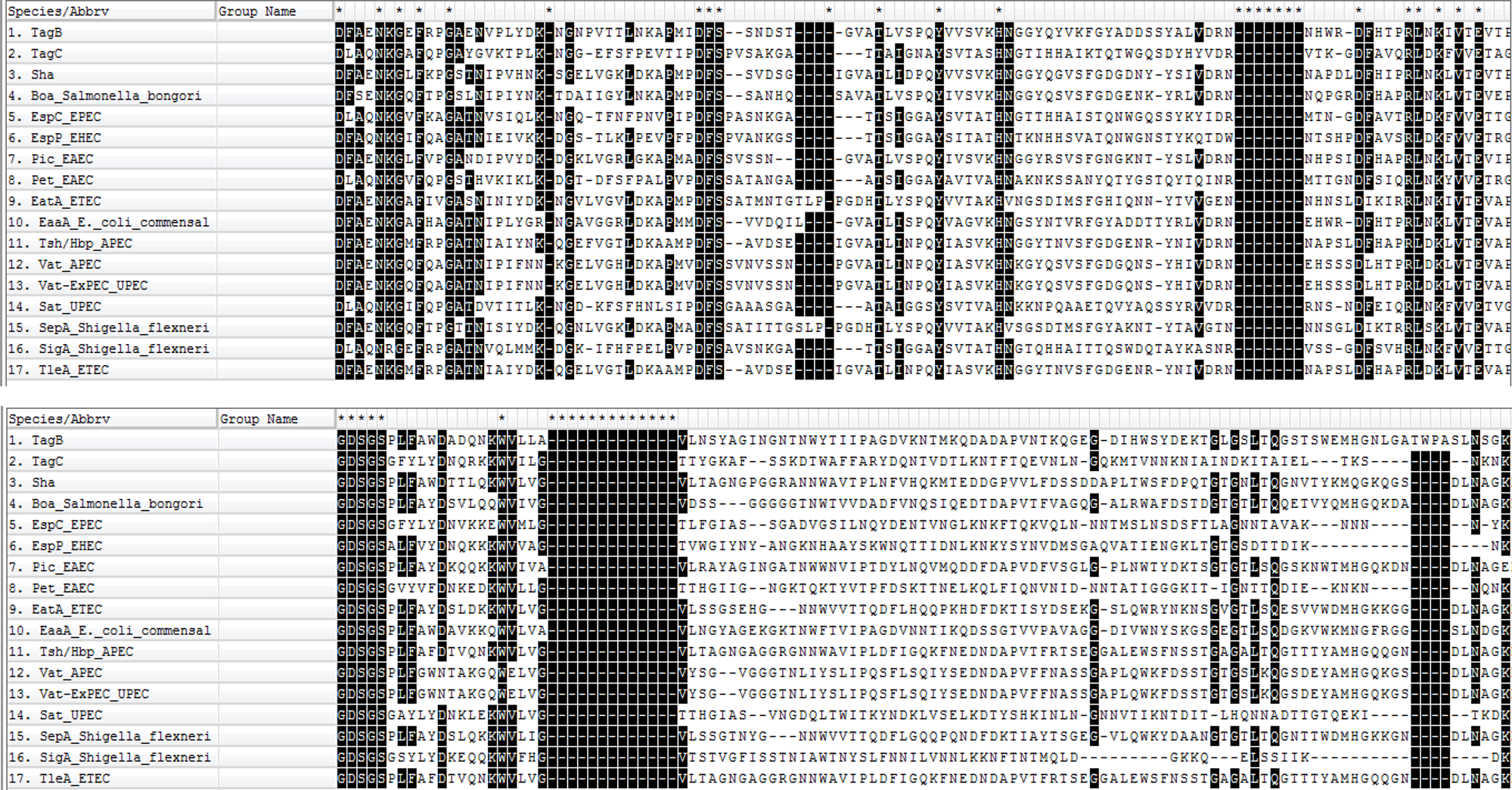

Supplement: Supplementary file 1 [file ijms-21-03047-s001.zip › Supplement Figure S4 I.tif]
